# A Preliminary Study on Public Health Implications of Avian Tuberculosis in Selected Districts of the Oromia Region, Ethiopia

**DOI:** 10.1155/2021/6331599

**Published:** 2021-10-13

**Authors:** Tesfaye Debelu, Fufa Abunna, Gezahegne Mamo Kassa

**Affiliations:** ^1^College of Agriculture and Natural Resources, Salale University, Fiche, Ethiopia; ^2^College of Veterinary Medicine and Agriculture, Addis Ababa University, Bishoftu, Ethiopia

## Abstract

**Background:**

Avian tuberculosis is a zoonotic disease which remains a problem in extensive poultry production systems under which chickens scavenge for survival in unhygienic environments. *Methodology*. A cross-sectional study was conducted from November 2016 to June 2017 at high-land areas of Gerar Jarso and Ada'a and Boset districts located at mid and low altitudes of Oromia, Ethiopia, respectively, to assess the perception of farmers on the occurrence of avian tuberculosis in chickens and its public health implications using a semistructured questionnaire.

**Result:**

The study evidenced poor awareness of the farmers, as only 11% (10/91) of them had well-perceived occurrence of the disease in chickens and its risk of zoonosis.

**Conclusion:**

Hence, it revealed that there is poor public perception on the occurrence as well as public health implications of avian tuberculosis, demanding further studies for verification and technical interventions.

## 1. Introduction

Avian tuberculosis is a list B disease of the World Organization for Animal Health caused by *Mycobacterium avium* or *Mycobacterium genavense*, which primarily affects poultry and captive birds [1]. It is also a public health threat, especially in immunocompromised individuals such as HIV patients. Infected birds and contaminated water and soil are the main sources of infection, since the organisms can survive for several months in the environment [2].

*Mycobacterium avium*, which is the major cause of the disease in humans, is very resistant to disinfectant chemicals and environmental extremes. It can survive in soil for ≥4 years, in 3% hydrochloric acid for ≥2 hours, and in 2% sodium hydroxide for ≥30 minutes [3]. Moreover, persistence within flocks and human exposure is also associated with keeping older stocks without following adequate cleanliness and hygiene. Furthermore, maintaining birds closely confined under stressful conditions may provide favorable ways for the spread of the disease. Thus, the ability of the organism to persist in the environment for many years, especially in soil and litter, favors the disease transmission to a greater extent [4].

*Mycobacterium avium* complex (MAC) is the second most common cause of pulmonary infection following *Mycobacterium tuberculosis* [5]. In humans, *M. avium* is capable of inducing a progressive disease that is refractory to antimicrobial therapy and is recognized as localized primary lymphadenitis, pulmonary disease, and a disseminated form of infection [6]. Hence, the handling of infected birds in farms or live cultures of *M. avium* in laboratories should be accomplished with adequate care [7].

On the other hand, in recent years, the incidence of avian tuberculosis in humans has shown an increasing tendency as human and bovine tuberculosis has begun to be eradicated in developed countries. This trend was also certainly affected by the development of diagnostic methods with better sensitivity and specificity. Apart from immunocompromised individuals, MAC is isolated increasingly from immunocompetent individuals [8].

The most common and well-recognized MAC infection in humans is a cavitary lung disease, predominantly involving the upper lobes, similar to the pulmonary TB seen in sanitarium patients [9, 10]. Patients with MAC lung disease tend to be older than TB patients and are not infectious for other humans [11]. Another relatively common form of disease caused by MAC is peripheral lymphadenopathy, which mainly affects children [12]. Lymphonodal infection is typically an infantile disease affecting, in most cases unilaterally, cervical lymph nodes [11]. The route of infection is most likely oral, and the childhood habit of bringing hands and objects to the mouth may well explain the particular susceptibility of infants to this pathology [13].

Disseminated MAC infections develop predominantly in severely immunocompromised people [14]. Among others, *M. avium* causes a serious disseminated infection in up to 40% of patients with advanced HIV infection. In AIDS patients, the main route for *M. avium* infection is the gastrointestinal tract, and *M. avium* is naturally tolerant to the low pH that exists in the stomach [15]. The transmission occurring via aerosols results in pulmonary infections as the organism frequently affects the lungs with endobronchial lesions. During the infection, *M. avium* can be demonstrated *in vivo* in the lymph nodes, bone marrow, urine, and sputum [16]. Primarily, the serotype-1 of *M. avium* subsp. *Avium* has been isolated from such individuals, clearly pointing the role of birds in acquiring infection [17]. It is also noteworthy that *M. avium* is a pathogen that infects several hosts including birds, humans, cattle, and pigs. They are also encountered in environmental sources such as soil and water, having considerable ability to overcome adverse and competitive conditions [18].

A study conducted at Shashemene district of Oromia, Ethiopia, indicated that, for chickens kept in an extensive production system, there exists a close physical contact between the chickens and their owners and there could be a possibility of transmission of *Mycobacterium avium* between chickens and their owners. On top of that, the low perception of the owners about zoonotic TB including avian TB and the high number of HIV patients and immunocompromised individuals in the country could add up to the risk of transmission [19].

In Ethiopia, although more than 95% of chickens are produced under a backyard poultry production system, where there are poor hygienic standards, strong physical contact between chickens and their owners and high risk of acquiring zoonotic diseases such as avian TB, studies conducted on avian tuberculosis are very scant. Hence, the objective of this study was to generate additional information on the public health implications of avian tuberculosis that helps to design appropriate control strategies of the disease.

## 2. Materials and Methods

### 2.1. Study Areas

The study was conducted from November 2016 to June 2017 at three selected districts of Oromia, Ethiopia, namely, Gerar Jarso, Ada'a, and Boset, located at high, mid, and low altitudes, respectively. From the three agroecologies in the region, one district was purposively selected and their respective peasant associations were selected based on chicken population and accessibility to roads.

Gerar Jarso district is located in the north Shewa zone of the Oromia regional state, 114 km north of Addis Ababa at 8.54°–10.23°N and 37.56°–39.24°E. The total area of the district is 42,763 hectares, of which 52%, 41%, and 7% are highland, midland, and lowland, respectively. The minimum altitude of the district is 1080 m, and the maximum is 3541 m above sea level. The average minimum and maximum annual rainfall are 793 mm and 1443 mm, respectively, while the average minimum temperature is 10°C and the maximum temperature is 32°C. The total population of the district is 67,298 (34,462 males and 32,836 females). There are 99,000 chickens in the district of which 84,000 (84.8%) are local and 15,000 (15.2%) are exotic breeds. [20]. Hence, for this study, PAs in highland areas of Gerar Jarso were selected.

Ada'a district is found 47 km southeast of Addis Ababa, the capital of Ethiopia. About 90% of the district belongs to the subtropical agroclimatic zone having an altitude ranging from 1500 to over 2000 m above sea level. The district receives an annual rainfall of 851 mm with annual minimum and maximum temperatures of 11 and 29°C, respectively. Although the district is most known for cereal crops (mainly teff and wheat) and legumes, livestock production is an integral part of the system. Cattle, small ruminants, poultry, and equines are the major livestock species kept in fast growing smallholder dairy production. The district has a total livestock population of 264,310 and a total poultry population of 107,554; of which 72,541 are exotic and the remaining 16,700 and 18,313 are local and cross breeds, respectively [21].

Boset is one of districts in the Oromia region of Ethiopia. It is part of east Shewa Zone located in Great Rift Valley. The district is bordered on the south by Arsi Zone, on the west by the Awash River which separates it from Adama, on the north by the Amhara Region, and on the east by Fentale. The administrative center of the district is Welenchiti. This district is predominantly level land with undulating features; almost 90% is less than 1500 meters above sea level; Boset Guddo is the highest point. A survey of the land in this district shows that 26.2% is arable or cultivable, 30% pasture, 15.8% forest, and the remaining 28% is considered barren, degraded, or otherwise unusable. Fruits and vegetables are important cash crops of the district. Boset has 97 kilometers of dry weather and 103 of all-weather roads, for an average of road density of 136.8 kilometers per 1000 square kilometers. About 87% of the urban, 36% of the rural, and 45% of the total population have access to drinking water. The 2007 national census reported a total human population of 142,112, of whom 73,925 were men and 68,187 were women; 26,514 or 18.66% of its population were urban dwellers [22]. The total chicken population of Boset district is 107,095, of which 95,070 are local and 12,025 are exotic [23].

### 2.2. Study Design and the Sampling Methodology

A cross-sectional study design was used primarily to determine the public health implications of avian tuberculosis in selected districts of the Oromia region, Ethiopia. Three districts, namely, Gerar Jarso, Ada'a, and Boset, were purposively selected from the high, mid, and low altitudes of the region, respectively. Moreover, a multistage sampling method was used to identify peasant associations (PAs) within the districts and households in the PAs. Thus, from each district, three PAs were selected based on their chicken population and road accessibility; then, 10 farmers who had chickens and willing to participate in the study were selected from different villages of each PA. A sampling frame of households who possess chickens was established with the help of livestock development agents. The unit of sampling was an individual household.

### 2.3. Sample Size Determination

A total of 91 households, which were proportionally divided among the districts, were purposively selected from the three study districts.

### 2.4. Questionnaire Survey

Ninety-one farmers in the three selected study districts, who were keeping scavenging chickens, were interviewed using a semistructured questionnaire to assess perceptions of the owners on the occurrence of avian tuberculosis in chickens and the public health implications of the disease.

### 2.5. Data Analysis

Descriptive statistics and Fisher's exact test were performed to analyze the data using Stata version 13.0 software package and Statistical Package for Social Sciences (SPSS) version 20.0. For all analyses that were performed, 95% CI and *P* value <0.05 was set for statistical significance of an estimate.

## 3. Results

### 3.1. Public Health Implications of Avian Tuberculosis

From a total of 91 chicken owners, 53 males and 38 females, interviewed to investigate the perception of the farmers on the occurrence of avian tuberculosis in chickens and its public health implications, only 11% (10/91) had the awareness of the disease and its zoonotic significance. Hence, the remaining 89% (81/91) of them had no awareness of the disease and its public health consequences.

**Figure 1:**
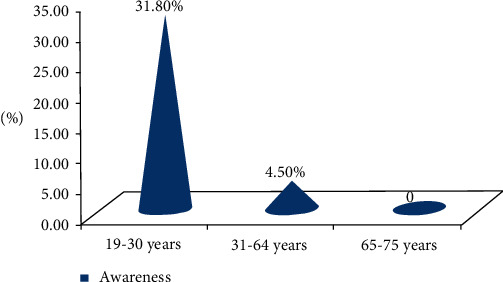
The trend of awareness in different age groups of farmers on the occurrence and public health implications of avian tuberculosis.

There was a significant awareness difference among farmers of different age groups in general, apart from the smaller proportion of people responded to have better perception of the disease and its zoonotic impact. In general, better awareness was observed in younger age groups between 19 to 30 years. Nonetheless, a significant awareness difference was not observed (*P* > 0.05) among farmers at different educational level, study districts, and the two gender categories involved in the study (Table 1 and Figure 1).

The data were also analyzed using Fisher's exact test to evaluate the experience of participant farmers on chicken housing and chicken house cleaning activities, in relation to their perception on avian TB and its zoonotic implications. Accordingly, 46.2% (36/91) of the chicken owners responded that they were keeping their chickens in a separate house at night. However, only 14.3% (6/36) of them were aware of avian TB and its zoonotic risks. In addition, a larger proportion of the participants responded their experience of sharing their home with chickens by keeping them on a perch or a carton in their home at night. Hence, the results revealed that there was no significant association (*P* > 0.05) between chicken housing, house cleaning, and farmers' awareness of the disease and its public health implications (Table 2).

## 4. Discussion

The present study attempted to investigate the public health implications of avian tuberculosis in selected districts of the Oromia region, Ethiopia. The results revealed that only 11% of the backyard chicken owners included in the survey responded that they had awareness on the occurrence of avian TB in chickens and its public health implications. However, 89% of them had no perception of both the occurrence and zoonotic significance of the disease; in spite of their gender, educational status, and spatial location. This result also agrees with the findings in [19] which reported the proportion of farmers who heard about avian TB and its zoonotic contribution as 13% and 2%, respectively, from Shashemane district of West Arsi, Ethiopia. On the other hand, apart from the smaller proportion of people responded to have better awareness of avian TB and its zoonotic implications, there was a significant (*P* > 0.05) awareness difference among farmers of different age groups, with better awareness observed in younger age groups between 19 to 30 years; probably due to the access to formal education.

This study clearly indicated that the lack of awareness of the majority of the farmers involved in the survey regarding the occurrence and public health implications of avian tuberculosis and hence its source of infection and mode of transmission. Conversely, according to the report in [24], in humans, all members of *M. avium* complex and *M. genavense* are capable of inducing a progressive disease that is refractory to treatment, mostly in immunocompromised patients. In addition, the situation worsens due to the spread of HIV infection in developing countries [16]. Therefore, it is considered prudent to keep infected birds away from humans, particularly the elderly, and individuals with poor immune status [25].

The study also assessed the experience of farmers participating in the study, on chicken housing and chicken house cleaning activities. Consequently, the results showed that 46.2% (36/91) of the participant farmers in the study were keeping their chickens in a separate house at night, and 22% (20/91) of them shared their home with chickens. However, there was no significant awareness difference (*P* > 0.05) among farmers of different age groups, gender categories, and at different educational levels and altitudes concerning chicken housing and house cleaning. The result of the current study also agrees with the findings in [26, 19] from different parts of Ethiopia which reported that most of the chicken owners share their homes with chickens, a situation which creates a conducive environment for the transmission of mycobacterial infection from chicken to humans. This multifaceted effect of lack of awareness and strong association of farmers with their chickens, especially in those farmers who share their home with chickens, could terribly predispose them to zoonotic diseases such as avian TB.

The other epidemiological risk factor that was noticed during this study which possibly favors the occurrence and chance of transmission of avian tuberculosis to humans was a situation in which farmers use the same night shade for chickens and domestic animals such as shoats and cattle. Consequently, 2.2% of the farmers involved in the survey responded that they were keeping chickens with shoats and the other 2.2% of them kept chickens with cattle in the same night shade, of which none of them had awareness of the disease and its public health implications. In such instances, there may be the risk of cross-transmission of the disease between chickens and other domestic animals, and hence, it becomes an immediate source of the disease for each other and humans as well. This finding also agrees with the findings in [26, 19], a study conducted at selected towns of central Ethiopia and Shashemene district of east West Arsi, which reported a large association of chickens with domestic animals and human beings which is stressful for the chicken and creates a conducive environment for the transmission of mycobacterial infection from chickens to other species including humans and vice versa. In addition, the study in [27] also identified contaminated food originating from pigs or other livestock as a potential source of human infection.

According to the view of participant farmers, consumption of chickens' meat and eggs was a common practice in the area and chicken slaughtering is commonly practiced at home. Nevertheless, the study in [28] reported that infected animals and their products, mainly eggs, which often come from small household production, are at risk for human health. Hence, in a community where the awareness of zoonotic diseases such as avian tuberculosis is poor and slaughtering is practiced under unhygienic conditions, these traditional practices could potentially affect the health of the public. This logic also holds true for urban dwellers in our country, Ethiopia, where chickens originated from the traditional chicken-producing areas of the country are extensively slaughtered at home, especially during major holidays such as Christmas, Easter, and Moulid, and it results in the highest risk of contamination with environmentally resistant bacterium, the cause of avian tuberculosis in birds and man. Hence, the situation urges for critical intervention programs to increase the level of perception of the society and device alternative chicken slaughtering methods.

## 5. Conclusions

The questionnaire survey to investigate the perception of farmers in the study area on the occurrence of avian tuberculosis in chickens and its public health implications evidenced poor awareness of farmers irrespective of their gender, educational status, and spatial location. Nevertheless, better awareness was observed in younger age groups of 19–30 years. However, in Ethiopia, where millions of chickens are reared by the rural population where the concept of hygiene and mode of disease transmission are underprivileged, the attention given to avian tuberculosis seems unrewarding. Therefore, based on the abovementioned conclusive remarks, the authors recommended that (1) medical professionals should give due consideration to the zoonotic risk of avian tuberculosis in diagnosing and treating multidrug resistant TB and extrapulmonary TB in human patients because tuberculosis due to *M. avium* is mostly intestinal form and hence extrapulmonary, which is also refractory to treatment. (2) The government should device a system which encourages chicken slaughter in the abattoir and discourages home slaughtering, which predisposes the public to the environmentally resistant etiology of ATB, particularly in developing countries where home slaughtering is a common practice both in rural and urban areas including major cities. (3) Attention should be given to awareness creation programs to traditional chicken producers and consumers concerning the predisposing factors of avian tuberculosis both in chickens and humans, especially in areas where the concept of hygiene and modes of disease transmission are poor.

## Figures and Tables

**Table 1:** Summary of the major host-related and environmental risk factors and their effect on the awareness of farmers involved in the study about the occurrence of avian tuberculosis and its zoonotic implications.

Variables	Category	Number of farmers participated in the study, % (N)	Number aware of avian TB in chickens and its zoonotic implications, % (N)	Fisher's exact value
Age	19–30 years	24.2 (22)	31.8 (7)	0.003
	31–64 years	72.5 (66)	4.5 (3)	
	65–75 years	3.3 (3)	0 (0)	

Gender	Male	41.8 (53)	7.5 (4)	0.310
	Female	58.2 (38)	15.8 (6)	

Edu. level	Illiterate	46.2 (42)	7.1 (3)	0.135
	Adult edu.	11 (10)	0 (0)	
	Elem. edu.	30.8 (28)	14.3 (4)	
	H. sch edu.	9.9 (9)	22.2 (2)	
	Co. edu.	2.2 (2)	50 (1)	

Altitude	Highland	33 (30)	20 (6)	0.119
	Midland	34.1 (31)	9.7 (3)	
	Lowland	33 (30)	3.3 (1)	

Edu. = education, Elem. = elementary, H. sch. = high school, and Co. = college.

**Table 2:** Summary of the fisher's exact test results on chicken housing and house cleaning experience of farmers participated in the study and their perception on avian TB and its zoonotic implications.

Variables	Categories	Number of farmers participated on the study % (N)	Number aware of avian TB in chickens and its zoonotic implications, % (N)	Fisher's exact value
Chicken housing	Separate	46.2 (36)	14.3 (6)	0.616
	W-human	22 (20)	15 (3)	
	W-shoats	2.2 (2)	0 (0)	
	W-cattle	2.2 (2)	0 (0)	
	On trees	27.5 (25)	4 (1)	

Chicken house cleaning interval	Daily	39.6 (36)	13.9 (5)	0.715
	E-2-3dd	24.2 (22)	9.1 (2)	
	Weekly	3.3 (3)	0 (0)	
	E-2-3 wks	3.3 (3)	33.3 (1)	
	Rarely	29.7 (27)	7.4 (2)	

W = with, E = every, dd = days.

## Data Availability

The data can be obtained from the corresponding author.
